# Heterogeneous Profile of ROR1 Protein Expression across Tumor Types

**DOI:** 10.3390/cancers16101874

**Published:** 2024-05-15

**Authors:** Maria Gabriela Raso, Elizve Barrientos Toro, Kurt Evans, Yasmeen Rizvi, Rossana Lazcano, Argun Akcakanat, Patrizia Sini, Francesca Trapani, Eva Johanna Madlener, Lorenz Waldmeier, Alexander Lazar, Funda Meric-Bernstam

**Affiliations:** 1 Department of Translational Molecular Pathology, The University of Texas MD Anderson Cancer Center, Houston, TX 77030, USA; enbarrientos@mdanderson.org (E.B.T.); rnlazcano@mdanderson.org (R.L.); 2 Department of Investigational Cancer Therapeutics, The University of Texas MD Anderson Cancer Center, Houston, TX 77030, USA; kwevans@mdanderson.org (K.E.); yqrizvi@mdanderson.org (Y.R.); aakcakanat@mdanderson.org (A.A.); fmeric@mdanderson.org (F.M.-B.); 3 Boehringer Ingelheim RCV, 1121 Vienna, Austriafrancesca.trapani@boehringer-ingelheim.com (F.T.); 4 Boehringer Ingelheim, 88400 Biberach, Germany; eva_johanna.madlener@boehringer-ingelheim.com; 5 NBE-Therapeutics AG, 4057 Basel, Switzerland; lorenz.waldmeier@boehringer-ingelheim.com; 6 Department of Pathology, The University of Texas MD Anderson Cancer Center, Houston, TX 77030, USA; alazar@mdanderson.org

**Keywords:** ROR1, tissue expression, IHC protein expression, tyrosine kinase-like orphan receptor

## Abstract

**Simple Summary:**

The research explores the potential of the Wnt receptor ROR1 as a target for cancer treatment, an area gaining significant attention. Despite ongoing therapeutic investigations, the understanding of ROR1’s presence in different tissues remains limited. To address this, the study conducted immunohistochemistry analyses on a large variety of tumor types, including sarcomas and carcinomas, revealing a diverse ROR1 expression pattern. Notably, high ROR1 prevalence was observed in mesothelioma, liposarcoma, gastrointestinal stromal tumors, and uterine endometrioid carcinoma. Conversely, other cancers showed lower expression. These findings highlight the potential for ROR1-targeted therapies, particularly in mesothelioma, which exhibits frequent and high ROR1 expression, suggesting a novel therapeutic avenue. This research may inform the development of more effective cancer treatments targeting ROR1.

**Abstract:**

The Wnt receptor ROR1 has generated increased interest as a cancer therapeutic target. Research on several therapeutic approaches involving this receptor is ongoing; however, ROR1 tissue expression remains understudied. We performed an immunohistochemistry analysis of ROR1 protein expression in a large cohort of multiple tumor and histologic types. We analyzed 12 anonymized multi-tumor tissue microarrays (TMAs), including mesothelioma, esophageal and upper gastrointestinal carcinomas, and uterine endometrioid carcinoma, among other tumor types. Additionally, we studied 5 different sarcoma types of TMAs and 6 patient-derived xenografts (PDX) TMAs developed from 19 different anatomic sites and tumor histologic types. A total of 1142 patient cases from different histologic types and 140 PDXs placed in TMAs were evaluated. Pathologists assessed the percentage of tumor cells in each case that were positive for ROR1 and the intensity of staining. For determining the prevalence of staining for each tumor type, a case was considered positive if >1% of its tumor cells showed ROR1 staining. Our immunohistochemistry assays revealed a heterogeneous ROR1 expression profile. A high prevalence of ROR1 expression was found in mesothelioma (84.6%), liposarcoma (36.1%), gastrointestinal stromal tumors (33.3%), and uterine endometrioid carcinoma (28.9%). Other histologic types such as breast, lung, renal cell, hepatocellular, urothelial carcinoma, and colon carcinomas; glioblastoma; cholangiocarcinoma; and leiomyosarcoma showed less ROR1 overall expression, ranging between 0.9 and 13%. No ROR1 expression was seen in mesenchymal chondrosarcoma, rhabdomyosarcoma, or gastric adenocarcinoma cases. Overall, ROR1 expression was relatively infrequent and low in most tumor types investigated; however, ROR1 expression was infrequent but high in selected tumor types, such as gastroesophageal GIST, suggesting that ROR1 prescreening may be preferable for those indications. Further, mesothelioma exhibited frequent and high levels of ROR1 expression, which represents a previously unrecognized therapeutic opportunity. These findings can contribute to the development of ROR1-targeted therapies.

## 1. Introduction

The tyrosine kinase-like orphan receptor 1 (ROR1) belongs to the receptor tyrosine kinase superfamily, which includes crucial regulators of normal cellular processes and cancer genesis [1]. The ROR subfamily members, ROR1 and ROR2, were designated as “orphan” receptors because their ligands were unknown for many years until it was discovered that they are associated with Wnt5A as its primary ligand [2,3].

ROR1 is essential during embryogenesis because it regulates cell migration, cell polarity, and organogenesis. In 1992, the gene encoding ROR1 was initially identified in neuroblastoma-derived cell lines [4]. ROR1 expression is associated with embryogenesis in the central nervous system, early limb bud, cartilage growth plate, heart, lung, and mesonephros in mice [5].

The WNT5A/ROR1 axis can activate several signaling pathways that are involved in cancer development, such as STAT3, PI3K/AKT, NF-κb, MAPK, and Hippo. There is evidence suggesting that the STAT3 pathway is an important target of ROR1 that can mediate the temporary effects of ROR1 expression in tumor cells and can also promote long-term effects through positive feedback pathways [6].

ROR1 has been reported to be abnormally expressed in malignancies and has been linked with poor survival and disease progression. These findings are likely due to ROR1’s role in supporting cell proliferation, survival, invasiveness, epithelial-to-mesenchymal transition (EMT), and metastasis [6]. ROR1 has also been shown to inhibit apoptosis, intensify epidermal growth factor receptor signaling, and participate in the epithelial-to-mesenchymal transition of tumoral cells [5]. Both ROR1 and ROR2 regulate β-catenin-independent signaling and have been associated with carcinogenesis and metastasis in several malignancies, including leukemias and lymphomas [7]. Balakrishnan et al. showed ROR1 expression in ovarian tumors, triple-negative breast carcinomas, and lung adenocarcinomas [8]. ROR1 and ROR2 were found to be absent in normal adult tissues in Western blotting studies. However, the same study states the possibility of ROR1 expression at low levels in the uterus, lung, bladder, testis, and colon [9]. Similarly, in other studies, ROR1 was found to be expressed at very low levels in human adult tissues, including the gastrointestinal and urogenital tract, testis, lung, parathyroid, and fibroblast-rich tissues. Conversely, higher expression levels were demonstrated in parathyroid, pancreas, and adipose tissues, raising concerns about potential toxic effects during targeted therapy [6,8,10]. We focused exclusively on ROR1 expression due to our interest in targeted therapy being developed for this specific target.

Increased expression of ROR1 is also associated with B-cell lymphocytic leukemia and a variety of other solid cancers [9,10,11,12]. These facts have made ROR1 a very attractive target for anticancer therapies, such as ROR1-targeted monoclonal antibodies, bispecific T-cell engagers, antibody–drug conjugates, chimeric antigen receptor T cells, and bispecific T-cell engagers, which are currently being evaluated in clinical trials [13,14,15,16,17,18,19,20].

Despite these advances, ROR1 tissue expression remains understudied. In this study, we performed a broad immunohistochemistry (IHC) analysis of ROR1 protein expression using a validated anti-ROR1 monoclonal antibody in several tumor types. This information will be fundamental in planning clinical trials for ROR1-targeted therapies.

## 2. Materials and Methods

### 2.1. Tissue Procurement

Protocols for the procurement of human tissues were approved by the Institutional Review Board (IRB) of the University of Texas MD Anderson Cancer Center. For the construction of multi-tumor and sarcoma tissue microarrays (TMAs), multiple malignant tumor types were selected from different tissue resources, including the MD Anderson Institutional Tumor Bank and the MD Anderson Department of Pathology. Donor blocks were sectioned, hematoxylin and eosin-stained slides were reviewed by pathologists, and specimens were selected based on tumor quantity and percentage of tumoral cells.

TMAs from patient-derived xenografts (PDXs) were made using 140 PDXs that were generated using human tumor samples from 19 different anatomical sites. Samples were collected with MD Anderson IRB approval. Tumors were then implanted in the mid-dorsal site of immunosuppressed (nude) mice. After tumors reached 1.5 cm in size, mice were euthanized, and the tumors were excised and fixed for the preparation of formalin-fixed paraffin-embedded PDX blocks. All animal experiments (protocol #00001405-RN01) were approved by MD Anderson’s Animal Care and Use Committee, which is accredited by AAALAC (Association for Assessment and Accreditation of Laboratory Animal Care) International. After hematoxylin and eosin-stained slides were reviewed by pathologists, specimens were selected based on tumor quantity and the absence of necrosis, fibrosis, or tissue folds. Control tissues were provided by FT and LW, and a single TMA composed of 5 different PDX tissue samples with known ROR1 expression was used as the positive and negative control for all IHC runs.

### 2.2. Tissue Microarray Preparation

For all TMAs, the core area selection was determined by quality control parameters, including the percentage of tumor cellularity content and the absence of necrosis and fibrosis. Multi-tumor and PDX TMA blocks were created using triplicates (three 1 mm cores selected from three equidistant points in the periphery, distal, and middle areas of each donor block) from a total of 792 cases. Sarcoma TMAs were built using duplicates (two 0.6 mm cores selected from the same tumor) from a total of 490 sarcoma cases.

### 2.3. Immunohistochemistry

We performed an automated IHC assay to detect ROR1 using a non-commercially available validated anti-ROR1 monoclonal antibody [8]. Recombinant cell culture-derived chimeric anti-ROR1 antibody clone 6D4 (mouse with rabbit constant regions; mab456) using a previously developed staining protocol for anti-ROR1 clone 6D4 (hybridoma) was validated by testing different primary antibody concentrations, antibody diluents, and blocking conditions. Applicability tests and assay adaptations were performed. The staining conditions were optimized to ensure adequate assay accuracy.

IHC was performed using a Leica BOND RXm Automated Stainer (Leica Biosystems, Nussloch, Germany). Formalin-fixed paraffin-embedded control tissues and multi-tumor, sarcoma, and PDX TMA slides were cut into 4-micron thickness and mounted on Superfrost Plus (Thermo Fischer, Waltham, MA 02451, USA and Indianapolis, IN 46250, USA) charged slides. Before loading, slides were baked at 60 °C for 1 h in a conventional oven. Leica BOND dewax solution was used for deparaffination, followed by EDTA buffer solution (pH 9.0 HIER2) for 20 min for heat-mediated epitope retrieval. All sections were treated with 3% hydrogen peroxide for 5 min to block endogenous peroxidase activity. Blocked sections were incubated with a monoclonal mouse antibody against human ROR1 as the primary antibody at a dilution of 0.5 µg/mL using Ventana Discovery antibody diluent (251-018, Roche Diagnostic, Indianapolis, IN 46250, USA) for 30 min. After primary antibody incubation, sections were incubated with bond polymer for 8 min. Antibody was detected using a BOND Polymer Detection Kit (DS9800; Leica Biosystems) with diaminobenzidine as the chromogen and counterstained with hematoxylin for 5 min.

After staining, the slides were washed with hot tap water, then placed in distilled water for a few seconds, and dehydrated using an ascending ethanol series, twice with 80% ethanol, twice with 96% ethanol, followed by a single wash of 100% ethanol; 1 min each; transferred to xylene (twice; 1 min each); and cover-slipped with mounting media (Cytoseal XYL catalog # REF-8312-4, Thermo-Fischer Scientific, USA). Appropriate positive, negative (diluent only), and isotype controls were stained in each IHC run.

### 2.4. IHC Analysis

Hematoxylin and eosin-stained TMA slides and ROR1 IHC-stained TMA slides were digitalized using an Aperio AT2 scanner (Leica Biosystems, Vesta, CA 92081, USA) under 20× objective magnification and evaluated by a pathologist. Analysis using bright-field microscopy was performed by pathologists; M.G.R. and E.B.T. scored the multi-tumor TMAs, M.G.R. and R.L. scored the sarcoma TMAs, and F.T. scored the PDX TMAs. The percentage of tumoral cells positive for ROR1 expression as well as H-scores were obtained based on the extent and intensity of membranous staining in tumor areas. For determining the prevalence of staining for each tumor type, a case was considered positive if >1% of its tumor cells showed ROR1 staining. Pathologists M.G.R., E.B.T., and F.T. performed interobserver comparisons.

## 3. Results

**Figure 1:**
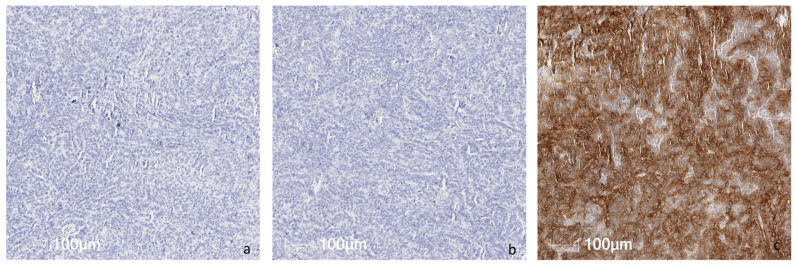
Representative pictures of endometroid carcinoma FFPE block as tissue control used for ROR1 IHC assay: (**a**) negative diluent, (**b**) negative isotype and (**c**) positive control.

**Figure 2:**
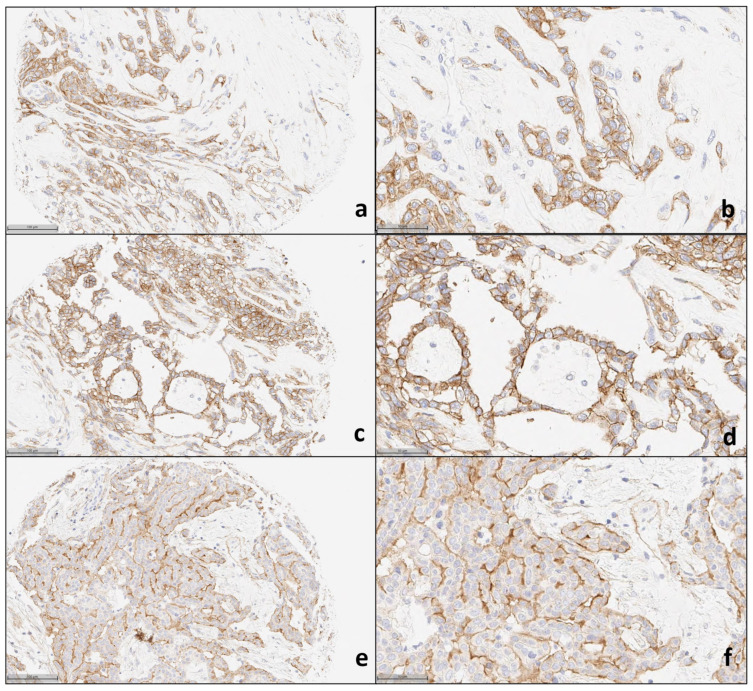
Representative pictures of ROR1 IHC expression in mesothelioma: 20 × magnification (**a**,**c**,**e**) and 40× magnification (**b**,**d**,**f**).

**Figure 3:**
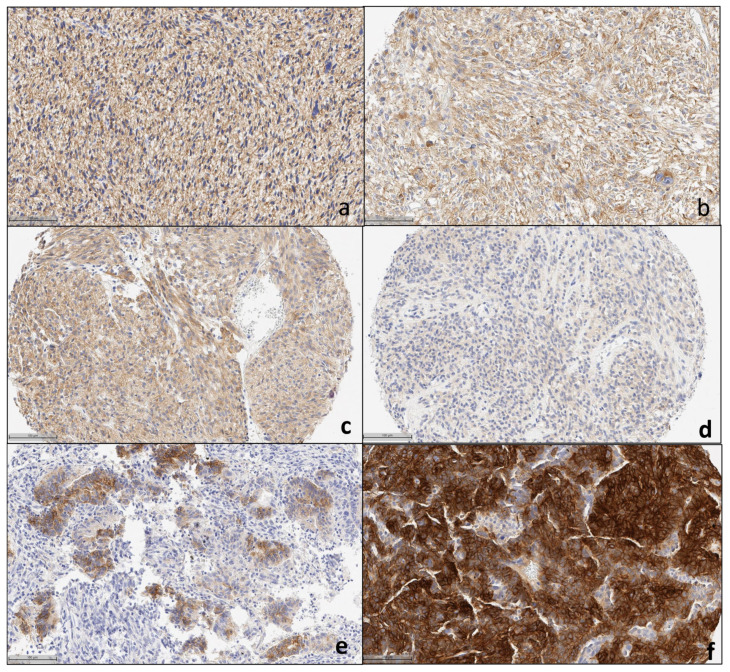
Representative pictures of ROR1 IHC expression in (**a**,**b**) liposarcoma, (**c**,**d**) GIST (gastrointestinal stromal tumor), (**e**,**f**) endometroid carcinoma, at 20× magnification.

**Figure 4:**
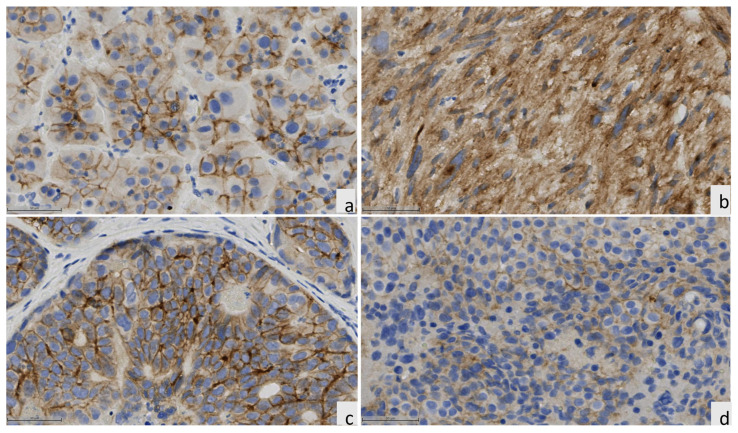
Representative pictures of ROR1 IHC expression in PDX (**a**) thyroid carcinoma. (**b**) sarcoma, (**c**) cholangiocarcinoma, and (**d**) pancreatic adenocarcinoma, at 20× magnification.

We evaluated 1142 patient cases from different histologic types and 140 PDXs placed in TMAs. The histologic characteristics of the multi-tumor TMAs, sarcoma TMAs, and PDX TMAs are detailed in Supplementary Tables S1–S3, respectively. Representative images of IHC staining for control tissues, patient tumor tissues, and PDX tumor samples are shown in Figure 1, Figure 2, Figure 3 and Figure 4.

**Figure 5:**
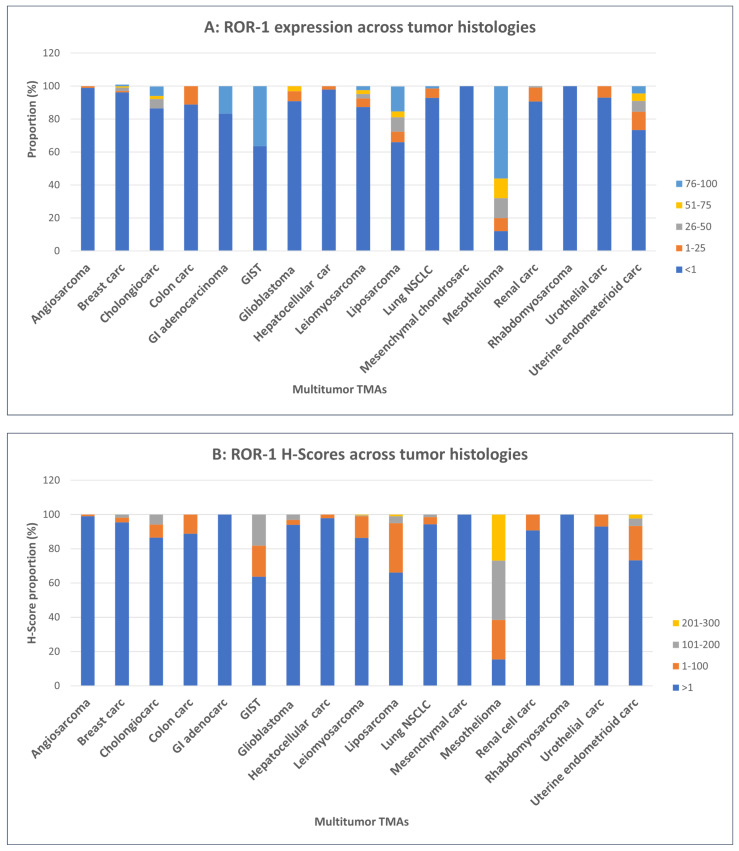
**(A) ROR1 expression and (B) H-score values across a multi-tumor TMA cohort.** Angiosarcoma (n = 102), breast (n = 105), cholangiocarcinoma (n = 52), colon adenocarcinoma (n = 45), GI adenocarcinoma (n = 24), GIST (n = 11), glioblastoma (n = 33), hepatocellular carcinoma (n = 47), leiomyosarcoma (n = 190), liposarcoma (n = 250), lung NSCLC (n = 70), mesenchymal chondrosarcoma (n = 08), mesothelioma (n = 26), renal (n = 108), rhabdomyosarcoma (n = 28), urothelial carcinoma (n = 43), and uterine endometrioid carcinoma (n = 45). Abbreviation: carc = carcinoma; chondrosarc = chondrosarcoma, NSCLC = non-small cell lung cancer.

**Figure 6:**
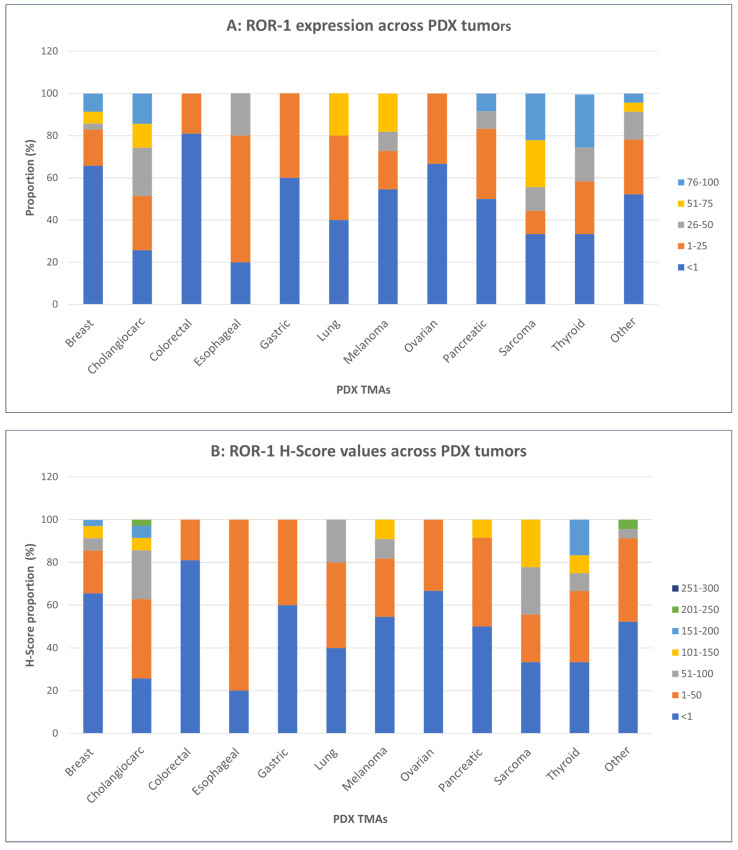
ROR-1 expression across PDX tumors: (**A**) ROR-1 percentage of expression and (**B**) H-scores values across PDX tumors by histology/location breast (n = 35), cholangiocarcinoma (n = 35), colorectal (n = 21), esophageal (n = 5), gastric (n = 5), lung (n = 5), melanoma (n = 11), ovarian (n = 6), pancreatic (n = 12), sarcoma (n = 9), thyroid (n = 12), other (n = 23).

The percentages of tumor cells positive for ROR1 expression in stained samples and the H-scores, incorporating stain intensity, are summarized in Figure 5 (patient tumors) and Figure 6 (PDX tumors).

**Figure 7:**
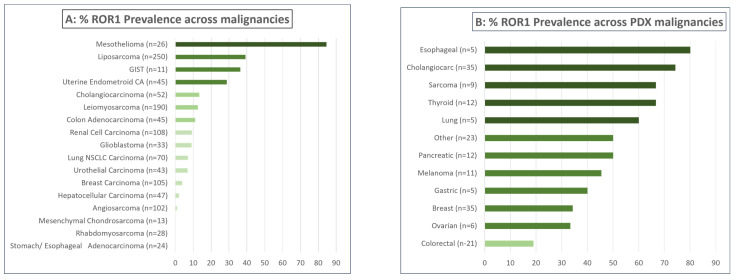
(**A**) ROR-1 prevalence across malignancies and (**B**) ROR-1 prevalence across PDX tumors.

The prevalence rates of ROR1 expression for each patient and PDX tumor type are summarized in Figure 7.

ROR1 was expressed in the membrane of tumoral cells, showing either partial or complete membrane staining, which was sporadically seen in the apical portion of the cells. Cytoplasmic staining was occasionally present. Intratumoral heterogeneity of expression was present in most tumor histologic types; however, liposarcoma cases exhibited consistently homogeneous ROR1 expression.

The average percentage of ROR1-expressing cells varied between histologic types, demonstrating higher expression in mesothelioma (average 68% expression) and liposarcoma (average 57% expression) to lower average expression levels (14–5%) in GIST, endometroid carcinoma, cholangiocarcinoma, and leiomyosarcoma. (Table 1, Figure 5A). Mesothelioma cases showed the highest intensity and H-scores compared to other tumor types. (Table 1, Figure 5B).

In our study, malignant mesothelioma showed the highest prevalence of ROR1 expression. Membrane/cytoplasmic ROR1 expression in >1% of tumoral cells was seen in 84.6% (22/25) and 56% (14/25) when considering a cut-off value of 146 based on the median H-score (Table 1). The average percentage of ROR1 expression in tumoral cells was 68.3% (range 0–100%), and the average H-score was 143.08 (range 0–286, median 146) for all mesothelioma histologic variants combined. Of these, 64% (16 cases) were in the pleura, followed by 20% (5 cases) with a peritoneal site and 16% (4 cases) in other locations. The cohort comprised 18 epithelioid variant cases, 6 biphasic variant cases, and 1 sarcomatoid variant case. We found a similar percentage of expression and H-scores in both the epithelioid and biphasic variants. The sarcomatoid variant case showed higher ROR1 expression than the other mesothelioma variants. Figure 2 shows representative paired 20× and 40× magnification images of ROR1 IHC expression in mesothelioma cases.

The liposarcoma cohort was composed of 133 liposarcomas, including well-differentiated lipoma-like and poorly differentiated variants. Figure 3a,b shows representative pictures of ROR1 IHC staining. Of these malignancies, the prevalence of expression was 36.1% (48/133). ROR1 membranous or cytoplasmic expression was identified in liposarcoma tumoral cells with an average positivity of 57.2% of tumor cells and an average H-score of 66.2. When comparing well differentiated versus poorly differentiated variants, no significant difference was noted.

Among the 12 gastrointestinal stromal tumor (GIST) cases, we found that 33.3% showed ROR1 expression, with an average total ROR1 positivity in tumor areas of 14% (range 0–100%) and an average H-score of 38 (range 0–160). Figure 3c,d shows representative GIST specimens ROR1 IHC expression.

Among the 45 uterine endometrioid carcinoma cases, we found that 28.9% showed ROR1 expression, with an average total ROR1 positivity in tumor areas of 9.9% (range 0–100%) and an average H-score of 16.8 (range 0–246). Figure 3e,f shows a representative sample. Supplementary Figures S1–S3 show representative high-definition detailed images.

Histologic findings from other tumor types showed low overall ROR1 expression that ranged between 0.98% and 13.5% of cases (Table 1). Gastric adenocarcinoma, rhabdomyosarcoma, and mesenchymal chondrosarcoma did not show ROR1 expression.

Our PDX TMA series was a heterogeneous group of PDXs comprising different histologic types (Supplementary Table S3). IHC staining demonstrated that thyroid carcinoma, sarcoma, and cholangiocarcinoma cases showed higher ROR1 prevalence and higher H-scores than other tumor types (Figure 6A,B). Overall, PDX histologic types showed similar patterns of ROR1 expression as the patients’ tumor types already discussed.

## 4. Discussion

ROR1 is currently being evaluated as a novel therapeutic target in many clinical trials using a variety of cell surface-targeting strategies. However, at least partially due to the lack of available commercial reagents, few studies have been conducted regarding the prevalence of ROR1 expression across tumor types. In our study, we found that the prevalence of ROR1 expression was low in most tumor types; however, a few tumor types, most notably mesothelioma, showed a large percentage of cancer cells expressed ROR1 and had a relatively high intensity of ROR1 expression.

To our knowledge, our study is the first publication to address the prevalence of ROR1 expression in malignant mesothelioma cases. We found that 84.6% of mesothelioma tumors studied were positive for expression of ROR1 when any expression was considered (cut-off > 1). Moreover, a high prevalence of 56% was noted when the median H-score cut-off was taken into consideration.

Saji et al. first demonstrated the presence of ROR1 and ROR2 protein expression in malignant pleural mesothelioma using IHC in five resected specimens, finding these proteins to be present in nearby noninvasive dysplastic mesothelial cells and absent in normal lung tissues [21]. Additionally, they examined the expression of ROR1 and ROR2 in human malignant pleural mesothelioma cell lines, finding higher ROR1 expression in H2452 cells than in other human lung mesothelioma cells. These findings suggest that ROR1 and ROR2 might be involved in the initiation and progression of malignant pleural mesothelioma associated with chronic inflammation and that these receptors could be novel molecular markers for malignant pleural mesothelioma. Our findings confirm this and suggest that ROR1 may be a novel therapeutic target for mesothelioma.

We also found notable ROR1 expression in other tumor types. Liposarcoma, the second most frequent soft tissue malignancy [22], is a mesenchymal tumor composed of mature adipocytes and stromal cells that account for up to 35% of all sarcoma types. It is histologically diverse [23], displaying a variety of genetic alterations, clinical behaviors, and prognostic courses [24]. ROR1 expression data in high-risk sarcoma, including Ewing sarcoma, osteosarcoma, alveolar or embryonal rhabdomyosarcoma, and fibrosarcoma, have been published [25,26], but none was found for liposarcoma.

GIST is a mesenchymal neoplasm of the gastrointestinal tract, usually located in the stomach and small intestine, but it can also arise in the colon, rectum, and esophagus [27]. Liver metastases are found concurrently with a primary GIST in up to 50% of cases [28]. Higher-risk GIST presents with liver metastasis 2 years after diagnosis in 40% to 80% of cases [28]. These tumors frequently have activating mutations in the KIT proto-oncogene that encodes a receptor tyrosine kinase or the PDGFRA gene that encodes platelet-derived growth factor receptor alpha [29]. The introduction of tyrosine kinase inhibitors (TKI) in clinical practice has significantly improved its prognosis, however, not all patients respond to TKI. Investigating novel and unexplored biomarkers, such as ROR1, may help select the most effective targeted drug in the future.

Endometrial cancer is the most prevalent gynecologic cancer and the sixth most common malignant tumor worldwide [30]. It is classified by its histopathologic characteristics as endometrioid adenocarcinoma, serous carcinoma, mucinous carcinoma, clear-cell carcinoma, or mixed carcinoma. These types are molecularly heterogeneous [31]. Our multi-tumor TMA cohort includes one TMA composed exclusively of endometrioid carcinoma cases. Microscopically, it resembles a proliferative endometrium, with a variable degree of glandular complexity and cellular pleomorphism [32,33]. A broad range of ROR1 and ROR2 expression levels were demonstrated in this tumor type by Liu et al., 2020 [34].

In addition to testing TMAs containing patient tumor samples, we also tested TMAs from PDXs containing multiple tumor types. The expression pattern in PDXs was also heterogeneous, but notably, expression was higher overall than in the patient specimens of corresponding tumor types. For example, only 13.5% of cholangiocarcinoma human tumors tested expressed ROR1, while 74% of cholangiocarcinoma-derived xenografts in our PDX TMAs expressed ROR1. These differences may be in part related to patient selection or due to the potential upregulation of ROR1 in more aggressive PDX tumors.

To date, several studies have reported that ROR1 is expressed in many tumor types, including breast cancer, ovarian cancer, and lung cancer [8,12,35]. Our findings differ from the literature, as we have found that expression of ROR1 is relatively infrequent. In addition, we found that when ROR1 is expressed, it is expressed at low intensity by a low percentage of cells. These findings are very relevant, as several ongoing clinical trials of ROR1-targeted therapies are not screening for ROR1 expression during patient selection. Importantly, we have found clinically meaningful ROR1 expression—highly frequent and moderately intense ROR1 expression—in mesothelioma and moderately frequent and lower intensity ROR1 expression—in liposarcoma. Noticeably, two other tumor types—gastrointestinal stromal tumors and endometroid carcinoma of the uterus demonstrated less frequent expression with lower intensities (Table 1). In summary, our findings may present ROR1-targeted therapies as novel therapeutic options for certain cancers.

Our study had two weaknesses. First, we used TMAs for screening, and we may have underestimated ROR1 expression in tumors that have heterogeneous expression. Of note, this could be the reason for the discrepancy with Balakrishnan et al.’s results regarding triple-negative breast carcinomas. Second, we tested over 1000 samples but did not test all tumor types reported to be positive for ROR1. Most notably, we did not test ROR1 expression in hematologic tumors; this is especially relevant in lymphoid cancers treated with zilovertamab vedotin, an antibody–drug conjugate targeting ROR1 that includes monomethyl auristatin E, a microtubule cytotoxin payload that demonstrated antitumor activity [36]. We plan to expand our cohort to include ovarian and endometrial serous carcinoma, as well as whole sections of the most prevalent tumor types, to evaluate tumor expression heterogeneity.

## 5. Conclusions

In conclusion, our study is the first to address the immunohistochemical ROR1 expression across several tumor types in a large sample cohort and to identify ROR1 prevalence in malignant mesothelioma. We found significant ROR1 expression in several tumor types, including mesothelioma, liposarcoma, GISTs, and endometrioid uterine carcinoma. These findings will contribute to the development of clinical trials involving ROR1-targeted therapies.

## Figures and Tables

**Table 1:** ROR1 expression in TMAs by tumor type. TMA, tissue microarray. Prevalence was defined as positive cases (those with >1% of tumor cells showing ROR1 expression) divided by total cases.

TMA	N	Average % Positive Cells	Average H-Score	Positive Cases (% Prevalence)
Mesothelioma	26	68.3	143	22 (84.6)
Liposarcoma	133	57.2	66.2	48 (36.1)
Gastrointestinal stromal tumors	12	14	38	4 (33.3)
Uterine endometrioid carcinoma	45	9.9	16.8	13 (28.9)
Cholangiocarcinoma	52	9.5	11.5	7 (13.5)
Leiomyosarcoma	213	5.3	6.15	27 (12.7)
Colon adenocarcinoma	45	0.5	0.79	5 (11.1)
Renal cell carcinoma	125	0.6	0.85	10 (8)
Non-small cell lung cancer	70	1.6	3.4	5 (7.1)
Urothelial carcinoma	43	0.1	0.16	3 (7)
Glioblastoma	33	2.3	3.9	2 (6.1)
Breast carcinoma	143	2.3	3.6	5 (3.1)
Hepatocellular carcinoma	47	0.1	0.32	1 (2.1)
Angiosarcoma	102	0.6	1.9	1 (0.98)
Gastric adenocarcinoma	11	0	0	0 (0)
Rhabdomyosarcoma	29	0	0	0 (0)
Mesenchymal chondrosarcoma	13	0	0	0 (0)

## Data Availability

The data generated in this study are governed by all participating institutions. To remain compliant with each participating institution’s regulatory requirements, aggregate and/or summary deidentified data may be made available upon reasonable academic request to the corresponding author.

## Supplementary Materials

The following supporting information can be downloaded at: https://www.mdpi.com/article/10.3390/cancers16101874/s1, Figure S1: Representative picture of ROR1 IHC expression in Mesothelioma; Figure S2: Representative picture of ROR1 IHC expression in Liposarcoma; Figure S3: Representative picture of ROR1 IHC expression in GIST; Figure S4: Representative picture of ROR1 IHC expression in Endometroid Adenocarcinoma. Table S1: Histologic types and variants of patient Multitumor TMAs studied; Table S2: Histologic features of patient sarcoma TMAs; Table S3: Histologic features of PDX TMAs.
