# Supplementary material for: Heterogeneous Profile of ROR1 Protein Expression across Tumor Types
Source: Cancers (Basel). 2024 May 15;16(10):1874. doi: 10.3390/cancers16101874 (PMC11119314; doi:10.3390/cancers16101874)
Supplement: Supplementary file 1 [file cancers-16-01874-s001.zip › Raso et al ROR1 paper Supplementary Tables vfinal clean.pdf]

Supplementary Table S1. Histologic types and variants of patient Multitumor TMAs studied.

TMA ID	Main histologic variant	Other Histologic variant
**Bladder**	Urothelial carcinoma NST	UC, Sarcomatoid<br>UC, Micropapillary<br>UC, Papillary
**Brain**	Glioblastoma	
**Breast**	IDC NST	Clear-cell carcinoma<br>Secretory carcinoma<br>ILC classical<br>ILC pleomorphic<br>Metaplastic<br>Mixed IDC/ILC
**Cholangiocarcinoma**	Cholangiocarcinoma	
**Colon**	Adenocarcinoma	Mucinous<br>NE/SCC<br>SRC
**Esophagus and stomach**	Adenocarcinoma	GIST

**Hepatocellular carcinoma**	Hepatocellular carcinoma	
**Kidney**	RCC	RCC, papillary<br>RCC, chromophobe
**Lung**	LUAD	Lung, SCC<br>Large cell carcinoma<br>Lung, LCNEC<br>NSCLC
**Mesothelioma**	Mesothelioma, epithelioid	Mesothelioma, biphasic<br>Mesothelioma, sarcomatoid
**Uterine endometrioid**	Endometroid carcinoma	

GIST, gastrointestinal stromal tumor; IDC, invasive ductal carcinoma; ILC, invasive lobular carcinoma; LCNEC, large-cell neuroendocrine carcinoma; NE, neuroendocrine; NST, no special type; RCC, renal cell carcinoma; LUAD, lung adenocarcinoma; SCC, squamous cell carcinoma; SRC; TMA, tissue microarray; UC, urothelial carcinoma.

Supplementary Table S2. Histologic features of patient sarcoma TMAs.

TMA ID	Main histologic type	Other Histologic variant
**Liposarcoma**	Liposarcoma	Well differentiated<br>Poorly differentiated
**Angiosarcoma**	Angiosarcoma	
**Leiomyosarcoma**	Leiomyosarcoma	
**Mesenchymal chondrosarcoma**	Mesenchymal chondrosarcoma	
**Rhabdomyosarcoma**	Rhabdomyosarcoma	

TMA, tissue microarray.

Supplementary Table S3. Histologic features of PDX TMAs.

TMA ID	Main histologic type	Other Histologic variant
**Breast**	Invasive breast carcinoma	Ductal<br>Lobular<br>Metaplastic
**Cholangiocarcinoma**	Cholangiocarcinoma	
**HER2-positive Tumors**	Any Histology HER2 positive cases	Breast carcinoma<br>Colorectal carcinoma<br>CCA<br>Other
**HER2 Negative tumors**	Any Histology HER2 negative cases	Thyroid carcinoma<br>Sarcoma<br>CCA<br>Other

CCA, cholangiocarcinoma; TMA, tissue microarray.
