# Supplementary material for: Heterogeneous Profile of ROR1 Protein Expression across Tumor Types
Source: Cancers (Basel). 2024 May 15;16(10):1874. doi: 10.3390/cancers16101874 (PMC11119314; doi:10.3390/cancers16101874)
Supplement: Supplementary file 1 [file cancers-16-01874-s001.zip › Supplementaty figures Raso et al Cancers 05142024 .pptx]

## Slide 1
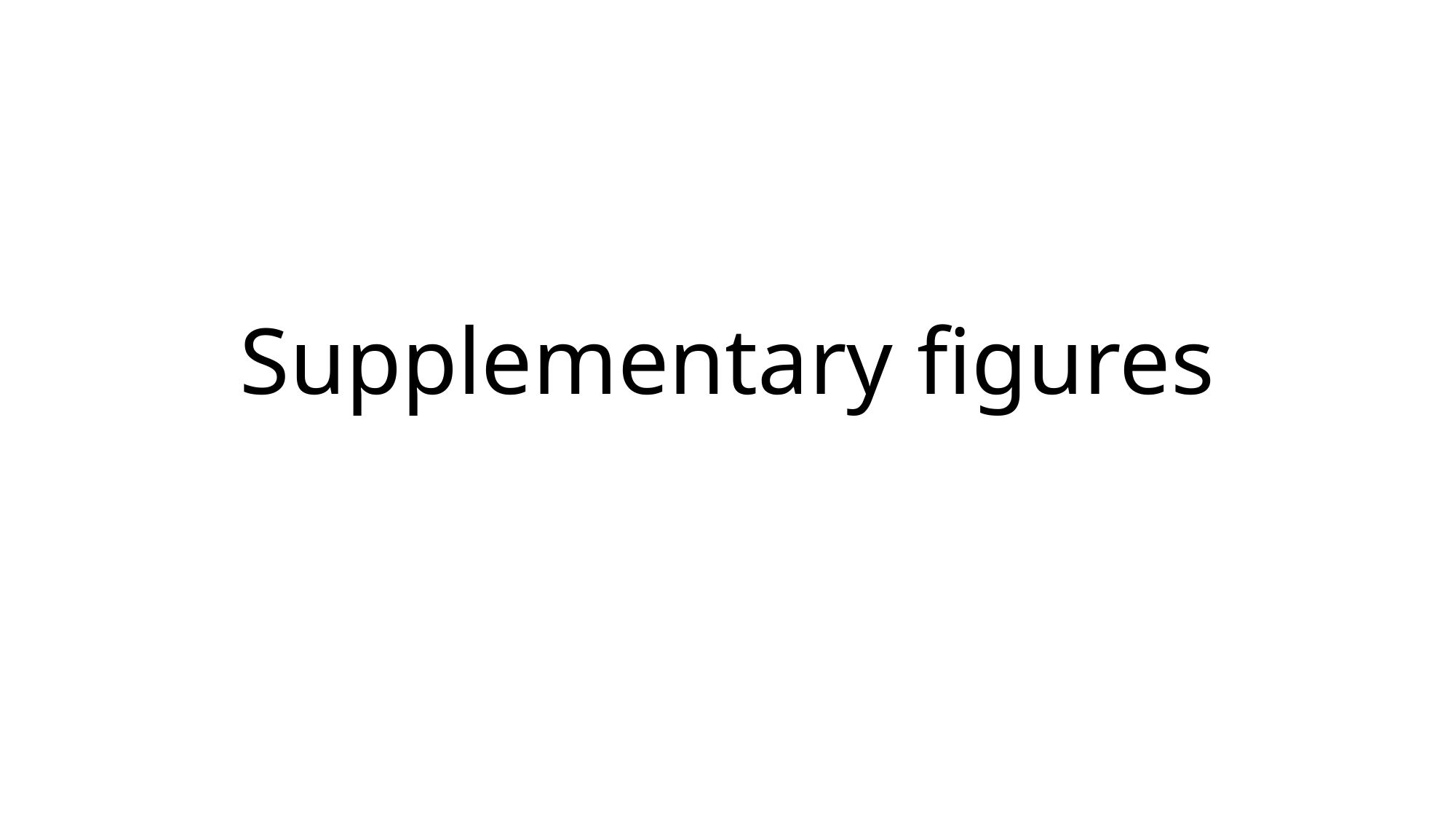

# Supplementary figures

## Slide 2
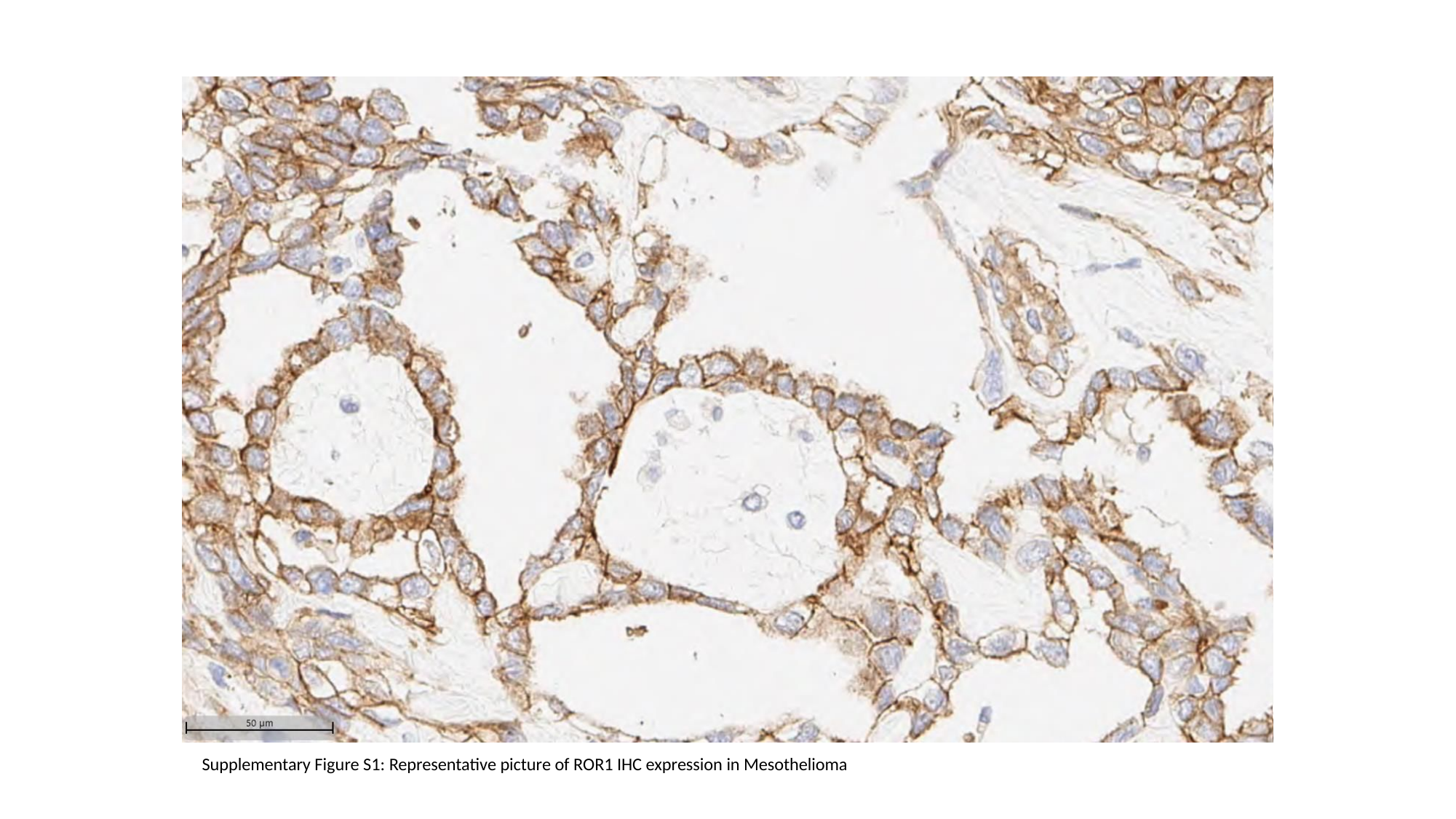

Supplementary Figure S1: Representative picture of ROR1 IHC expression in Mesothelioma

## Slide 3
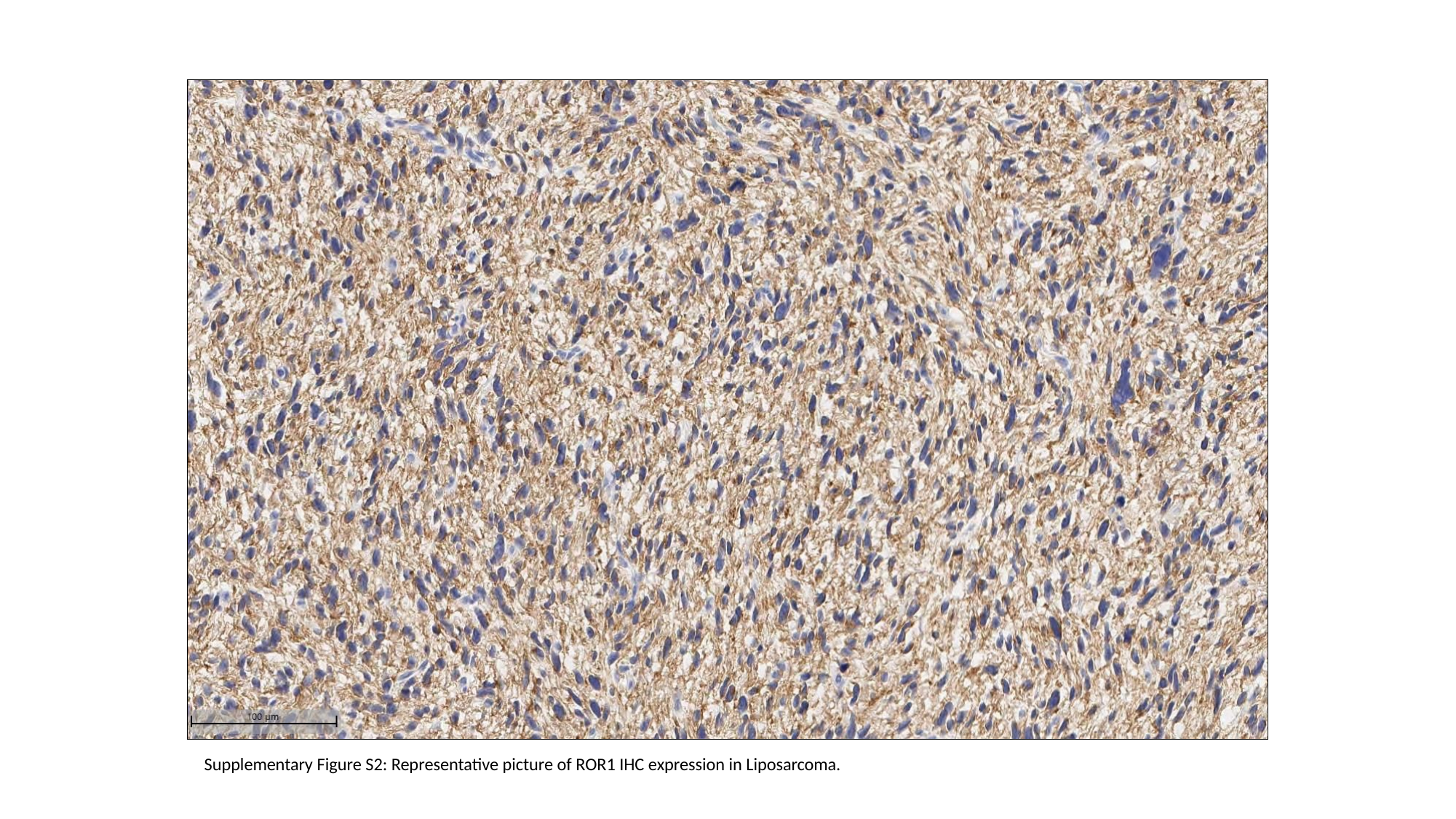

Supplementary Figure S2: Representative picture of ROR1 IHC expression in Liposarcoma.

## Slide 4
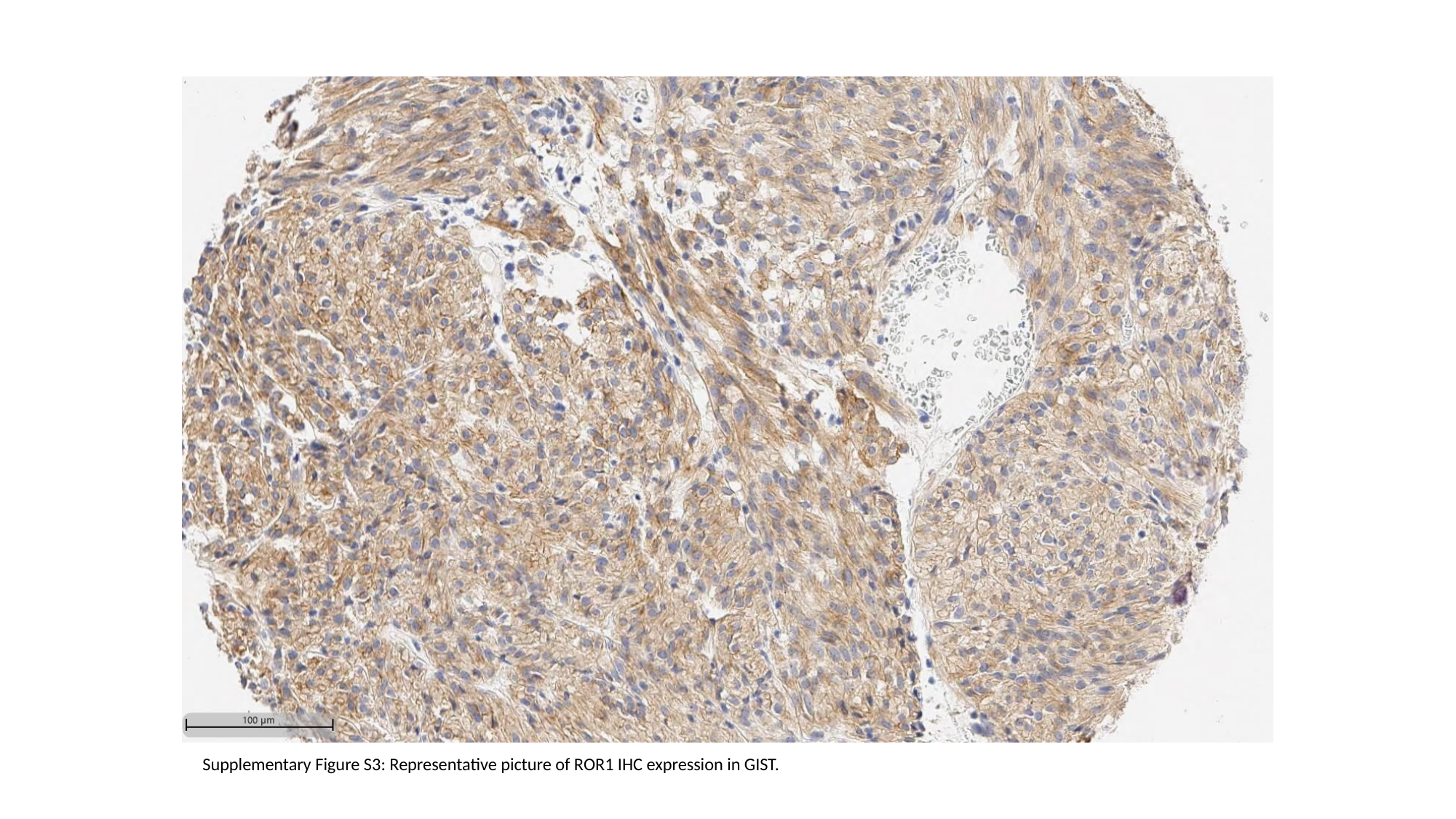

Supplementary Figure S3: Representative picture of ROR1 IHC expression in GIST.

## Slide 5
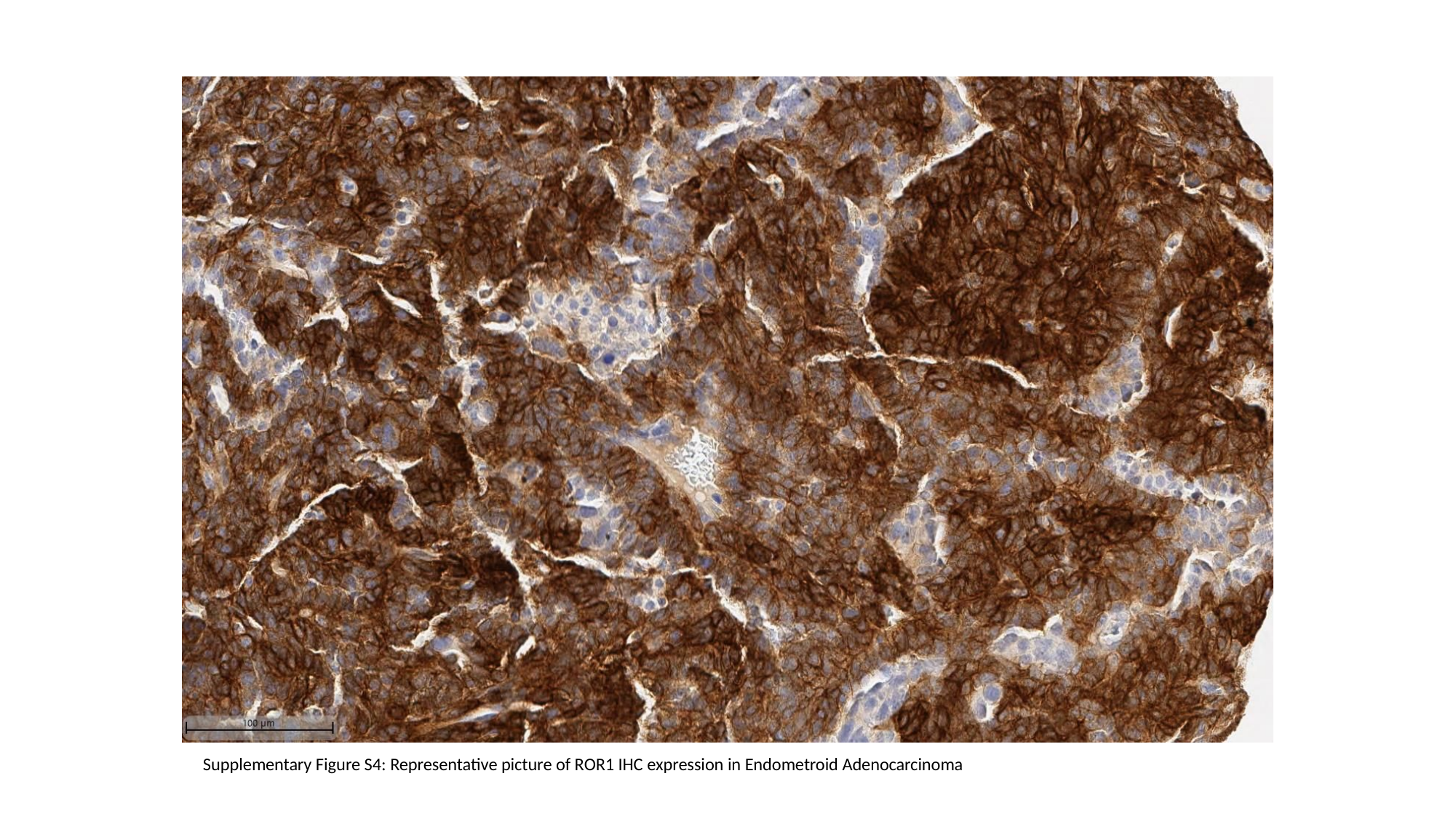

Supplementary Figure S4: Representative picture of ROR1 IHC expression in Endometroid Adenocarcinoma
